# Incidence and Antimicrobial Sensitivity Profiles of Normal Conjunctiva Bacterial Flora in the Central Area of China: A Hospital-Based Study

**DOI:** 10.3389/fphys.2017.00363

**Published:** 2017-05-31

**Authors:** Hua Tao, Juan Wang, Lei Li, Hui-Zhi Zhang, Meng-Ping Chen, Le Li

**Affiliations:** ^1^Department of Pharmacy, Zhengzhou Second HospitalZhengzhou, China; ^2^Management Office of Science and Education, Zhengzhou Second HospitalZhengzhou, China; ^3^Department of Ophthalmology, Zhengzhou Second HospitalZhengzhou, China; ^4^Department of Clinical Laboratory, Zhengzhou Second HospitalZhengzhou, China

**Keywords:** normal conjunctival, sac-bacterial, culture-antimicrobial susceptibility test, micro-organisms, Chinese

## Abstract

**Objective:** To study the distribution and patterns of resistance to antimicrobial agents of normal conjunctival bacteria.

**Materials and Methods:** Conjunctival specimens were collected from 8,224 patients and then cultured, which underwent antimicrobial susceptibility test following standard methods. Patients with infectious symptoms such as erythema or oedema and those using systemic or topical antibiotics within 1 month were excluded.

**Results:** In this study, the incidence of isolated bacteria was 24.2%. The middle aged group of 41–65 years presented the lowest rate of bacterial isolation which was 19.4%, while the highest isolation rate (83.1%) was found in patients in the age range of 0–6 years. In every age group, the incidence of bacterial isolation in men was higher than that in women. The top 3 most commonly isolated micro-organisms were *Staphylococcus epidermidis* (39.7%), *Streptococcus pneumoniae* (4.5%), and *Staphylococcus aureus* (2.7%), of which about 83.1% *S. aureus* were isolated in the group of 0-6 years. We found that *coagulase-negative Staphylococcus* (CONS) were more resistant to penicillin, macrolides, clindamycin and sulfonamides with the rate ranging from 57.9 to 90.8%, which were highly susceptible to vancomycin, linezolid, rifampin, tetracyclines, and aminoglycosides. Contrasting to CONS, the general resistance rate of *S. aureus* was significantly lower. Additionally, *Streptococcus* was susceptible well to the majority of antimicrobial agents, while highly resistant to macrolides and tetracyclines with the rate >80%.

**Conclusions:** In conclusion, our study revealed the incidence and antimicrobial sensitivity profiles of normal conjunctiva bacterial flora in the central area of China, which could be useful in the prevention of ocular infections. Importantly, our data could be used to guide the selection of appropriate prophylactic agents.

## Introduction

Conjunctival sac is colonized by a diverse range of microorganisms which constitute the normal ocular flora or potential pathogens. However, these microorganisms are capable of causing infections of conjunctiva and cornea when changes happen to microenvironment of the ocular surface or systemic condition. Especially when the globe of the eye is breached by trauma or surgery, these floras could be the predisposing factors of the endophthalmitis (Armstrong, 2000; Deorukhkar et al., 2012). Due to multiple environments as well as different ethnic groups and patterns of administration, the global epidemiology indicates that the spectrum of conjunctival sacs pathogens and resistance patterns vary between geographic locations (Bertino, 2009; Passos et al., 2010). Moreover, reports of microbial isolates from either diseased or preoperative ocular tissues varied widely according to published studies (Khristov, 1956; Sharma et al., 2013).

This study was carried out in Chinese population of substantial size, which seeked to define the incidence and antimicrobial sensitivity profiles of normal conjunctival bacterial flora in the central area of China. Since eye care professionals regularly provide antibiotic prophylaxis to their patients following ocular trauma/injury, such data could be used to guide the selection of appropriate prophylactic agents. Thus, our study could be useful in the prevention of ocular infections and in choosing the initial empirical therapy prior to final laboratory results.

## Methods

### Study subjects

A total of 8,224 patients presenting to the Second People's Hospital of Zhengzhou for refraction, cataract surgery, or other clearance surgeries, were selected for this research with consent from ethics committee. The patients were divided into 5 groups: children (0–6 years), adolescence (7–17 years), young (18–40 years), middle aged (41–65 years), elderly (>65 years). The protocol of this study was approved by the ethics committee of the Second People's Hospital of Zhengzhou in accordance with the Declaration of Helsinki.

### Ocular and systemic examination

Detailed ocular and systemic examination was conducted to rule out the presence of surface infection or an ocular disease with erythema or oedema. Other exclusion criteria were topical antibiotic use within 1 month and known patients with a systemic illness or an immunodeficiency disease. Only one eye of a patient was randomly selected for the study. Conjunctival anesthesia was done by instilling 1–2 drops of 0.5% proxymetacaine and a sample was collected by gently rubbing the conjunctiva of lower fornix from medial to lateral side with a sterilized cotton-wool swab stick moistened with a drop of sterile normal saline, taking care not to touch the lid margins and ensuring that individuals should not blink during the procedure. The samples were inoculated into enrichment broth plate, and then were placed in the thermostat bacterial incubator at 35°C for 24, 48, and 72 h. Plate was examined for bacterial growth. Colonies of positive samples were immediately transferred into the blood agar plate, incubated at 35°C for 24–48 h, and then the colonies were identified after isolation and purification.

### The antimicrobial susceptibility testing

The specimens were inoculated to blood agar plate and chocolate plate, incubated at 35°C, 5% carbon dioxide for 24 h. Appropriate detection reagents were selected according to the gram staining and morphology of specimens. The antimicrobial susceptibility test was conducted according to the Clinical and Laboratory Standards Institute (CLSI) standard 2012. TDR-300B bacterial identification and drug sensitivity tester and automatic microbiological analysis system were used to detect the antimicrobial susceptibility of the tested strains with the broth dilution method. Quality control strains were used as follows: ATCC29213 *Staphylococcus aureus*, ATCC49619 *Streptococcus pneumoniae*, ATCC49247 *Haemophilus influenzae*, ATCC27853 *Pseudomonas aeruginosa*, ATCC25922 *Escherichia coli*, and ATCC29212 *Enterococcus faecalis*.

### Statistical analysis

Collected data were analyzed using the SPSS13.0, and a descriptive analysis was conducted to clarify the isolation rate, the resistance and susceptibility to the antimicrobial agents. The Chi square statistic was carried out to compare the isolation incidence of the patients with different ages and gender. To evaluate the association between the incidence of tested germ and the age of patients, we undertook a stratified analysis with Chi square statistic after stratifying the information by gender. We also adopted Chi square statistic to analyze the difference between resistance of coagulase-negative Staphylococcus (CONS) and resistance of *S. aureus* to all kinds of antimicrobial agents.

## Results

**Figure 1:**
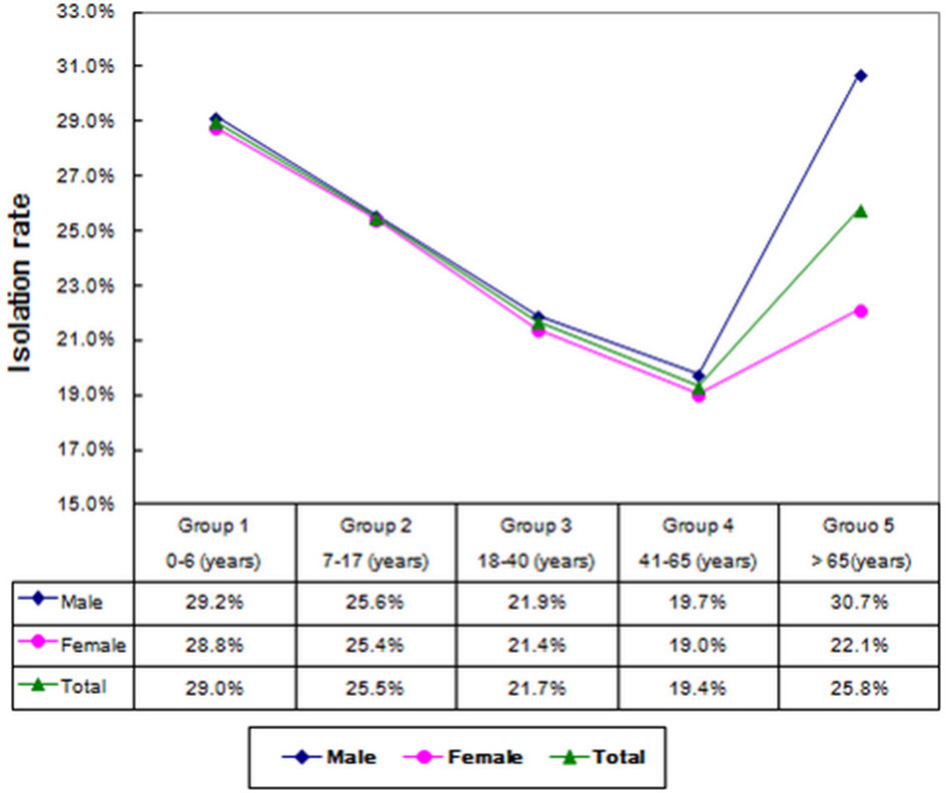
The isolation rates of conjunctival bacterial among different age groups.

A total of 8,224 conjunctival samples collected from 4,266 males (51.9%) and 3,958 females (48.1%) were divided into 5 groups (Table 1). The median age of the patients was 37.0 ± 28.2 years and the age difference between males and females was significant (χ^2^ = 7.107, *P* = 0.008). The isolation rate in the age groups differed significantly (χ^2^ = 57.346, *P* < 0.01), namely, 29.0% (506/1,743) in 0–6 years group, 25.5% (377/1,478) in adolescence group, 21.7% (230/1,059) in young group, 19.4% (423/20,184) in the 41–65 years group, and 25.8% (454/1,760) in the elderly group. Age was an influencing factor on bacterial isolation in the patients with different gender. For example, the incidence of bacterial isolation in males (30.7%) was significantly higher than that in females (22.1%) in elderly group (χ^2^ = 16.674, *P* < 0.01). As shown in Figure 1, the incidence of bacterial isolation showed a trend of decreasing at first, and then climbed up along with age. The middle aged group of 41–65 years presented the lowest rate of bacterial isolation (19.4%), while the highest isolation rate (83.1%) was found in patients in the age group of 0–6 years. Furthermore, in every age group, the incidence of bacterial isolation was higher in men than that in women.

**Table 1:** The isolation rates of conjunctival samples in each group.

**Age (years)**	**Overall**		**Male**		**χ^2^**	***P***	**Female**		**χ^2^**	***P***
	**Samples (*n*)**	**Isolation (*n*)**	**Samples (*n*)**	**Isolation (*n*)**			**Samples (*n*)**	**Isolation (*n*)**		
0–6	1,743	506	993	290	39.904	<0.01	750	216	27.667	<0.01
7–17	1,478	377	892	228			586	149		
18–40	1,059	230	625	137			434	93		
41–65	2,184	423	1,008	199			1,176	224		
>65	1,760	454	748	230			1,012	224		

Of the 1,990 isolated strains, 920 (46.2%) strains were only gram stained without performing specific strain identification and susceptibility test for the following reasons: 887 strains were gram-positive corynebacterium, and the majority were normal flora of human skin and mucosa, which needed to be further identified only in special circumstances (Wang et al., 2015); 33 strains of gram-positive cocci were also not identified. In spite of these unclear isolations above, we eventually isolated 1,070 strains and 24 types of flora. The number of isolation types of flora was 19 in children group, 10 in adolescence group, 10 in young group, 8 in middle aged, and 12 in elderly group, respectively. The most commonly isolated bacteria was *S. epidermidis* (39.7%, 790/1,990), surpassing *S. pneumoniae* (4.5%, 89/1,990) which was followed by *S. aureus* (2.7%, 54/1,990) and *Moraxella catarrhalis* (2.2%, 44/1,990). The isolation rates exhibited statistically significant difference in each age group (Table 2).

**Table 2:** Proportion of bacterial isolates from conjunctival specimens.

**Micro-organism**	**Group 1**	**Group 2**	**Group 3**	**Group 4**	**Group 5**	**Total**	**χ^2^**
	**0–6 years**	**7–17 years**	**18–40 years**	**41–65 years**	**>65 years**		
	***n* = 506(%)**	***n* = 377 (%)**	***n* = 230(%)**	***n* = 423(%)**	***n* = 454(%)**	***n* = 1070(%)**	
**GRAM-POSITIVE BACTERIA**							
*S. epidermidis*	117 (23.1)	157 (41.6)	137 (59.6)	204 (48.2)	175 (38.5)	790 (39.7)	109.699^*^
*S. pneumoniae*	74 (14.6)	6 (1.6)	4 (1.7)	1 (0.2)	4 (0.9)	89 (4.5)	164.844^*^
*S. aureus*	13 (2.6)	21 (5.6)	4 (1.7)	10 (2.4)	6 (1.3)	54 (2.7)	16.049^*^
*S. hominis*	6 (1.2)	6 (1.6)	6 (2.6)	10 (2.4)	3 (0.7)	31 (1.6)	6.291
*S. haemolyticus*	3 (0.3)	2 (0.5)	2 (0.9)	2 (0.5)	5 (1.1)	14 (0.7)	–
Others	9 (1.8)	0 (0)	1 (0.4)	1 (0.2)	3 (0.7)	14 (0.7)	–
**GRAM-NEGATIVE BACTERIA**							
*M. catarrhalis*	33 (6.5)	6 (1.6)	1 (0.4)	1 (0.2)	3 (0.7)	44 (2.2)	60.187^*^
Others	13 (2.6)	5 (1.3)	3 (1.3)	2 (0.5)	9 (2.0)	32 (1.6)	7.125
Fungus	1 (0.2)	1 (0.3)	0 (0)	0 (0)	0 (0)	2 (0.1)	–
Total	269 (53.2)	204 (54.1)	158 (68.7)	231 (54.6)	208 (45.8)	1,070 (53.8)	32.328^*^

* *P < 0.01*.

**Figure 2:**
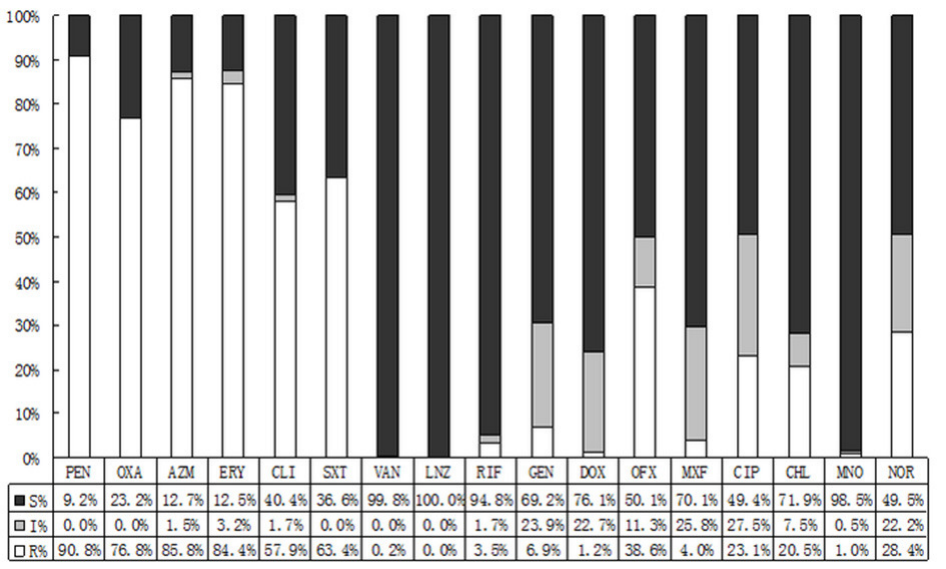
The antibiotic resistance patterns of coagulase-negative Staphylococcus (CONS) strains. S, Sensitive; I, Intermediate; R, Resistant; PEN, Penicillin; OXA, Oxacillin; AZM, Azithromycin; ERY, Erythromycin; CLI, Clindamycin; SXT, Trimethoprim-Sulfamethoxazole; VAN, Vancomycin; LNZ, Linezolide; RIF, Rifampicin; GEN, Gentamycin; DOX, Doxycycline; OFX, Ofloxacin; MXF, Moxifloxacin; CIP, Ciprofloxacin.

The susceptibility profiles of different isolates also differed with type. CONS was the most frequently isolated flora from the conjunctival sacs. Most CONS were resistant to penicillin (90.8%), followed by azithromycin (85.8%), erythromycin (84.4%), oxacillin (63.4%), and clindamycin (57.9%). Except moxifloxacin (4.0%), the resistance rates for CONS to quinolones were observed between 23.1 and 38.6%. Besides, CONS were susceptible well to a spectrum of antibacterial agents such as rifampicin (3.5%), minocycline (1.0%), gentamycin (6.9%), and doxycycline (1.2%). As shown in Figure 2, we discovered that 2 species of CONS were resistant to vancomycin, and no strain of CONS was resistant to linezolid.

**Figure 3:**
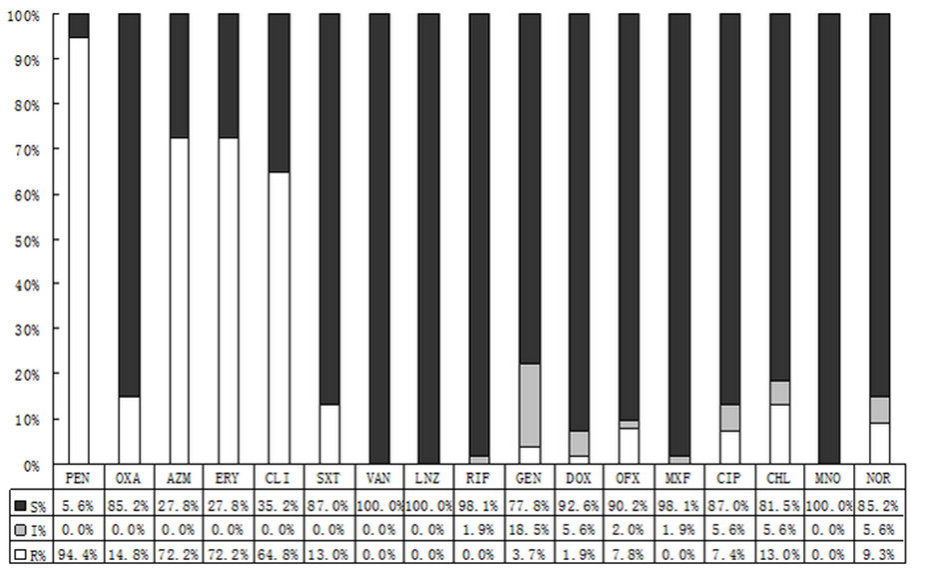
The antibiotic resistance patterns of *Staphylococcus aureus* (*S. aureus*) strains. S, Sensitive; I, Intermediate; R, Resistant; PEN, Penicillin; OXA, Oxacillin; AZM, Azithromycin; ERY, Erythromycin; CLI, Clindamycin; SXT, Trimethoprim-Sulfamethoxazole; VAN, Vancomycin; LNZ, Linezolide; RIF, Rifampicin; GEN, Gentamycin; DOX, Doxycycline; OFX, Ofloxacin; MXF, Moxifloxacin; CIP, Ciprofloxacin.

By contrast with CONS, *S. aureus* demonstrated lower resistance rates. It performed like that: 76.8% of isolating CONS were resistant to oxacillin, while in *S. aureus* the rate was 14.8%. To co-trimoxazole, the resistance rates of CONS and *S. aureus* were 63.4 and 13.0%, respectively. And the resistance rates to azithromycin and erythromycin were 85.8 vs.72.7%, and 84.4 vs. 72.2%, respectively. All the differences were statistically significant. Comparing to CONS with resistance rate 4.0–38.6%, *S. aureus* showed lower resistance rate with 0.0–9.3% to quinolone. There was no resistant strain to vancomycin, linezolid, rifampicin, or minocycline among *S. aureus* (Figure 3).

**Figure 4:**
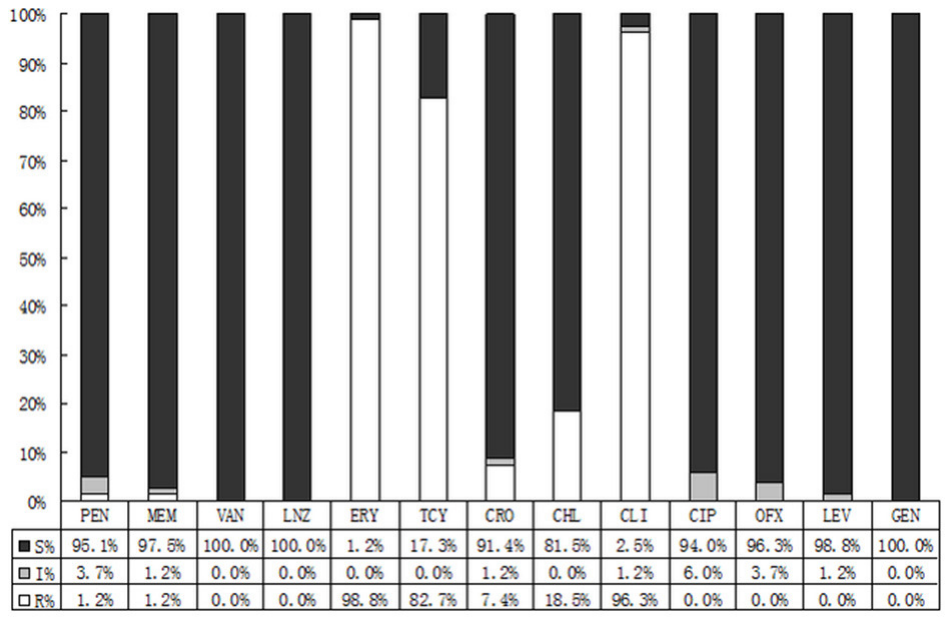
Results of sensitivity against *Streptococci* spp.antibiotics. S, Sensitive; I, Intermediate; R, Resistant; PEN, Penicillin; VAN, Vancomycin; LNZ, Linezolide; RIF, Rifampicin; ERY, Erythromycin; TCY, Tetracycline; CRO, Ceftriaxome; CLI, ClindamycinS; CIP, Ciprofloxacin; OFX, Ofloxacin; LEV, Levofloxacin.

As demonstrated in Figure 4, *Streptococcus* was sensitive to vancomycin, linezolid, and levofloxacin, whereas highly resistant to erythromycin (98.8%), clindamycin (96.3%), and tetracycline (82.7%).

## Discussion

The support of conjunctival sac on bacteria may result from the direct contact with the outside environment and the connection to the adjacent skin, and the different results of culture are greatly attributed to various factors such as environment, age, administration of antimicrobial agents, etc. (Barfoed, 1956; Khristov, 1956; Sharma et al., 2013). The use of antimicrobial agents contributes to the emergency of the new drug resistant strains (Leibovitch et al., 2005; Recchia et al., 2005), therefore, the pathogens of ocular infectious diseases are more attractive to the clinical researchers. But only few investigations have focused on the microflora of normal healthy conjunctival sacs. Our study analyzed the results of culture and antimicrobial susceptibility testing of 1,070 types of normal conjunctival flora, expecting to elaborate the diffusion and antimicrobial susceptibility of regional normal conjunctival flora, thereby making a contribution to the clinical recommendations for the prescription.

In this study, the overall incidence of isolated bacteria was 24.2%, lower than the published results of 36.0–81.7% (Ta et al., 2009; Sharma et al., 2013; Muluye et al., 2014). Previous publications reported that certain local anesthetic eye drops had strong antimicrobial effects, which were dose-related and related to whether the drops contained preservatives (Mullin and Rubinfeld, 1997; Dantas et al., 2000; Pelosini et al., 2009). Compared to published results, we detected a relatively lower incidence of conjunctival flora in present study, and the use of local anesthetic might be contributory. Twenty-four species of conjunctival micro-organism were isolated altogether, and the difference of bacterial isolation among various age groups was statistically significant. The isolated rate showed a trend of decreasing at first and then climbed up along with age. The middle aged group of 41–65 years presented the lowest rate of bacterial isolation (19.4%). Further investigation showed that in every age group, the incidence of bacterial isolation in men was higher than that in women, especially in elderly group (>65 years), and the difference was significant (Singer et al., 1988; Li and Smith, 2002).

The most commonly isolated micro-organism was *S. epidermidis* (39.7%), which was followed by *S. pneumoniae* (4.5%) and *S. aureus* (2.7%). *S. epidermidis* was the predominant isolate, that was consistent with previously reported trends (Ta et al., 2009; Sharma et al., 2013; Muluye et al., 2014). The majority (83.1%) of the bacterial isolates were from patients in the age range of 0–6 years. Gram negative isolates were found in small numbers only, with *M. catarrhalis* being most common (2.2%), and we also isolated other strains of gram negative rods such as *P. aeruginosa, Philo acinetobacter, Klebsiella pneumoniae*, and *Proteus mirabilis*.

It was thought that normal ocular flora could be non-pathogenic or occasionally pathogenic. However, the pathogens of some bacterial endophthalmitis, bacterial corneal ulcers, blepharitis, conjunctivitis, and other ocular infection diseases turned out to consistent with conjunctival isolated bacteria, and the *S. epidermidis* has become the predisposing pathogen (Recchia et al., 2005; Carreras, 2012; Sharma et al., 2013). Although, the preoperational topical antibiotics can hardly make the conjunctival sacs sterile, they can effectively reduce the isolating bacteria (Mshangila et al., 2013). The susceptibility of each antibiotic to different bacteria is not identical, and the regional reported drug resistance varies widely due to different environment and the use of antibiotics (Carreras, 2012; Mshangila et al., 2013; Muluye et al., 2014). The overall incidence of isolated bacteria was 24.2%, and we found higher resistance rates to penicillic, macrolides, clindamycin and sulfonamides by CONS, ranging from 57.9 to 90.8%. With a relatively high susceptibility to moxifloxacin, the CONS was moderately susceptible to other fluoroquinolones, and highly susceptible to vancomycin, linezolid, rifampin, tetracyclines, and aminoglycosides. Contrasting to CONS, the general resistance rate of *S. aureus* was lower. *Streptococcus* was susceptible well to the majority of antimicrobial agents, while highly resistant to macrolides and tetracyclines with the rates >80%.

Evolving bacterial resistance represents one of the most serious global public health problems, and overcoming this problem has become a great challenge. Due to different ethnic group, environment and antimicrobial therapy, the distribution and resistant profiles of conjunctival bacteria vary significantly from area to area. Therefore, the investigation in these subjects can be clinically useful in the primary empirical antimicrobial strategy before knowing the laboratory results.

## Author contributions

HT and LeiL conceived the study. LeiL and MC performed the laboratory experiments. JW and HZ contributed to the statistical analysis. HT wrote the draft of the paper. HT and LeL reviewed the manuscript. All authors have read and approved the final manuscript.

### Conflict of interest statement

The authors declare that the research was conducted in the absence of any commercial or financial relationships that could be construed as a potential conflict of interest. The reviewer AG and handling Editor declared their shared affiliation, and the handling Editor states that the process nevertheless met the standards of a fair and objective review.
